# Dietary bile acid supplementation alters plasma biochemical and hormone indicators, intestinal digestive capacity, and microbiota of piglets with normal birth weight and intrauterine growth retardation

**DOI:** 10.3389/fmicb.2022.1053128

**Published:** 2022-11-10

**Authors:** Yang Liu, Md. Abul Kalam Azad, Qian Zhu, Zugong Yu, Xiangfeng Kong

**Affiliations:** ^1^College of Veterinary Medicine, Nanjing Agricultural University, Nanjing, Jiangsu, China; ^2^Key Laboratory of Agro-ecological Processes in Subtropical Region, Key Laboratory of Animal Nutritional Physiology and Metabolic Process, National Engineering Laboratory for Pollution Control and Waste Utilization in Livestock and Poultry Production, Institute of Subtropical Agriculture, Chinese Academy of Sciences, Changsha, Hunan, China

**Keywords:** bile acid, digestive capacity, intestinal development, liver protection, microbial community, weaned piglet, intrauterine growth retardation

## Abstract

Piglets with intrauterine growth retardation (IUGR) have poor small intestinal morphology and function, resulting in impaired digestion and absorption of nutrients and lower growth performance. Bile acids (BA) are important in regulating digestive enzyme activity, digestion and absorption of lipids, intestinal development, and protecting the liver. The present study aimed to investigate the effects of dietary BA supplementation on plasma biochemical and hormone indicators, intestinal morphology and function, and microbial community in piglets with normal birth weight (NBW) and IUGR. Weaned piglets (24 IUGR and 24 NBW) were allocated to four groups (12 piglets per group) and fed the following diets: (i) NBW group, NBW piglets fed a basal diet; (ii) NBW + BA group, NBW piglets fed a basal diet with 400 mg/kg BA; (iii) IUGR group, IUGR piglets fed a basal diet; and (iv) IUGR + BA group, IUGR piglets fed a basal diet with 400 mg/kg BA. The feeding trial lasted 28 days. The results showed that IUGR decreased the weight of the jejunum, whereas dietary BA supplementation decreased the jejunum weight and increased the length, weight, and index of ileum in NBW piglets (*p* < 0.05). In addition, IUGR increased (*p* < 0.05) the plasma choline esterase (CHE) and glucose levels of weaned piglets regardless of BA supplementation. Dietary BA supplementation increased the plasma albumin, triglyceride, and total protein concentrations while decreased plasma aspartate transaminase (AST), alanine aminotransferase (ALT), CHE, lactate dehydrogenase, and NH_3_ levels regardless of IUGR (*p* < 0.05). The IUGR increased trypsin level in the ileum, whereas dietary BA supplementation decreased jejunal trypsin and lipase and ileal lipase levels of weaned piglets regardless of IUGR (*p* < 0.05). Spearman’s correlation analysis revealed the potential link between the intestinal microbial community and intestinal health-related indices of weaned piglets. These findings suggest that IUGR could decrease small intestinal morphology and function, whereas dietary BA supplementation could promote the ileum development of NBW piglets, protect the liver by reducing plasma ALT and AST levels, and increase the proportion of potentially beneficial bacteria in the small intestine of NBW and IUGR piglets, contributing to intestinal development and health of weaned piglets.

## Introduction

Intrauterine growth retardation (IUGR) becomes a major threat in animal production due to the harmful effects on postnatal intestinal health and growth performance (Longo et al., 2014). There are more than 15% of piglets suffer from IUGR at birth (Wu et al., 2006). Previous studies have reported that IUGR impaired the intestinal morphology and barrier function of neonatal piglets (Wang et al., 2008), which resulted in lower digestion and absorption of nutrients in the small intestine of IUGR piglets (Ferenc et al., 2014). Furthermore, the pig’s intestine harbors a diverse abundance of microbes, which play vital roles in intestinal development, homeostasis, and the maturation of the innate immune system (Jiang et al., 2019). A previous study indicated that IUGR could alter the diversity and abundance of intestinal microbiota, which was closely associated with neonatal piglets’ intestinal digestive and barrier function (Zhang et al., 2019). However, little information exists to overcome these poor situations of IUGR.

Bile acids (BA), derived from cholesterol in the hepatocyte, play vital roles in facilitating the digestion and absorption of nutrients (mainly lipids) and regulating the development of the small intestine (Wahlström et al., 2016). For example, BA can modulate the level of enzymes, which catalyzes the hydrolysis of cholesterol esters into free cholesterol and fatty acids, enabling their absorption by the intestinal mucosa (Lombardo, 2001). Research evidence showed that dietary BA supplementation could increase nutrient digestibility (Maisonnier et al., 2003) and growth performance and decrease abdominal fat deposition in poultry (Geng et al., 2022). Furthermore, BA have been found to inhibit the overgrowth of gut bacteria; however, the intestinal microbiota can regulate BA synthesis, increase the diversity of BA by bile salt hydrolase and steroid dehydrogenase, and produce secondary BA metabolites through deconjugation and dehydroxylation reactions (Cai et al., 2020). Previously we found that IUGR could alter the small intestinal structure and microbial community of weaned piglets (Zhang et al., 2019). Moreover, IUGR was also associated with the dysregulation of BA metabolism in growing piglets (Liu et al., 2022). However, the effects of dietary BA on the intestinal digestion and microbial community of weaned piglets remained unclear.

Therefore, we hypothesized that dietary BA supplementation might improve the small intestinal morphology and function by altering the microbial community of weaned piglets. Thus, the present study was conducted to determine the effects of dietary BA supplementation on digestive enzyme level, morphology and function, and microbial community of the small intestine of weaned piglets with normal birth weight (NBW) and IUGR. This study will provide a reference for BA application in swine production.

## Materials and methods

### Animals and experimental design

Forty-eight neonatal piglets at birth (one NBW and one IUGR piglet per litter; a total of 24 NBW and 24 IUGR piglets) were selected for this study. Based on our previous study (Xiong et al., 2020), piglets with the lowest birth weight were classified as IUGR piglets, and piglets with the highest birth weight per litter were classified as NBW piglets. Piglets were weaned at 21 days of age. After 7 days of adaptation, NBW (7.43 ± 0.18 kg) and IUGR (5.86 ± 0.20 kg) piglets were randomly allocated to one of four groups (*n* = 12 per group), including the NBW group (NBW piglets received a basal diet), NBW + BA group (NBW piglets received a basal diet with 400 mg/kg BA), IUGR group (IUGR piglets received a basal diet), and IUGR + BA group (IUGR piglets received a basal diet with 400 mg/kg BA). The composition and nutrient levels of basal diets met the National Research Council (NRC, 2012) recommendation (Supplementary Table S1). All weaned piglets were housed in a controlled temperature (23°C–25°C) and humidity (60 ± 5%) room and had free access to food and drinking water at all times. Piglets were fed three times per day at 8:00, 13:00, and 18:00 with their respective diets. The experiment lasted 28 days. The BA was provided by Longchang Animal Health Care Co., Ltd. (Shangdong, China). The purity of bile acid was ≥98.5%, derived from pig bile consisted chenodeoxycholic acid (17%), hyodeoxycholic acid (68%), and hyocholic acid (9%).

### Sample collection

All piglets were fasted for 12 h, weighed, and then euthanized by a jugular puncture after anesthesia (intravenous injection of 4% sodium pentobarbital solution) on day 28 of the experiment. The blood samples (5 ml) were withdrawn from the anterior vena cava of each piglet into heparin-treated tubes (Saihua, China). Plasma was obtained by centrifuging at 3,000 ×*g* for 10 min at 4°C and then stored at −20°C for biochemical analysis. The weight and length of the jejunum and ileum were recorded using the electronic scale and tape. The jejunum (10 cm below the duodenum-jejunum junction) and ileum (10 cm above the ileocecal junction) segments (approximately 1.5–2 cm) were excised, washed with phosphate-buffered saline (PBS) to remove intestinal contents, and then fixed with 4% paraformaldehyde to determine the morphology. Fresh small intestinal contents (~5 g) were collected into sterile tubes from the jejunum and ileum, immediately frozen in liquid nitrogen, and then stored at −80°C for microbial genomics total DNA extraction. The jejunal and ileal mucosa samples were excised, rinsed with ice-cold PBS, immediately frozen in liquid nitrogen, and then stored at −80°C for digestive enzyme level determination.

### Determination of plasma biochemical parameters

Plasma biochemical parameters, including albumin (ALB), glucose (GLU), high-density lipoprotein-cholesterol (HDL-C), low-density lipoprotein-cholesterol (LDL-C), NH_3_, total cholesterol (TC), triglyceride (TG), total BA (TBA), total protein (TP), and urea nitrogen (UN), as well as the levels of amylase (AMS), alanine aminotransferase (ALT), alkaline phosphatase (ALP), pancreatic amylase (AMY), aspartate transaminase (AST), choline esterase (CHE), and lactate dehydrogenase (LDH) were determined using the Cobas C311 Automatic Biochemical Analyzer (Cobas, Basel, Switzerland) and the respective commercial assay kits (Leadman Bio. Co., Ltd. Leadman, Beijing, China).

### Determination of plasma hormone concentrations

The plasma hormone concentrations, including cholecystokinin (CCK), gastrin (Gas), glucocorticoid (GC), ghrelin (Ghr), insulin-like growth factor 1 (IGF-1), insulin-like growth factor 2 (IGF-2), insulin (INS), leptin (LEP), pancreatic polypeptide (PP), peptide YY (PYY), and somatostatin (SS) were determined using porcine ELISA kits (Meimian, Wuhan, China) according to the manufacturer’s protocols. The absorption values were read on Multiscan Spectrum Spectrophotometer (Tecan, Infinite M200 Pro, Switzerland).

### Determination of the intestinal digestive enzyme levels

Approximately 100 mg of frozen jejunum and ileum mucosa samples were removed quickly, homogenized with ice-cold PBS (1:9, w/v), and then centrifuged at 2,000 ×*g* for 20 min at 4°C. The levels of amylase, trypsin, and lipase in the intestinal supernatants were determined using the porcine ELISA kits (Huyu, Shanghai, China), following the manufacturer’s instructions. The absorption values were read on Multiscan Spectrum Spectrophotometer (Tecan, Infinite M200 Pro, Switzerland). The enzyme levels were normalized to the total protein concentration quantified by the Pierce BCA Protein Assay Kit (Beyotime, Shanghai, China).

### Intestinal morphology analysis

Jejunal and ileal tissues fixed with 4% formaldehyde were embedded in paraffin, and sections (5 μm thickness) were obtained and stained with hematoxylin and eosin (H&E). Digital images of the intestinal morphology at 40 and 100 × magnification was obtained using a light microscope (Nikon, Japan). The villus height (VH; the distance from the crypt-villus junction to the tip of the villus) and crypt depth (CD; the distance from the bottom of the crypt to the crypt-villus junction) of 10 intact villus-crypt units were measured using Image-pro plus 6.0 (Media Cybernetics, Inc., Rockville, MD, United States), and the VH/CD ratio was calculated.

### Intestinal microbiota analysis

Total DNA was extracted from 200 mg of each jejunal and ileal contents using the QiaAmp Fast DNA Stool Mini Kit (Qiagen, Germantown, MD, United States) following the manufacturer’s instructions. The V3-V4 region of the 16S rRNA gene was amplified with universal primers 341F (5′-ACTCCTACGGGAGGCAGCA-3′) and 806R (5′-TCGGACTACHVGGGTWTCTAAT-3′). The amplified products were detected using agarose gel electrophoresis (2% agarose), recovered by AxyPrep DNA Gel Extraction Kit (Axygen Biosciences, Union City, CA, United States), and then quantified by a Qubit 2.0 Fluorometer (Thermo Fisher Scientific) to pool into equimolar amounts. Amplicon libraries were sequenced on an Illumina MiSeq 2,500 platform (Illumina, San Diego, CA, United States) for paired-end reads of 250 bp.

The Illumina platform was used to generate paired-end reads (2 × 300). Sequencing and bioinformatics analyses were performed on QIIME2 platform by the Shanghai Personal Biotechnology Co., Ltd. (Shanghai, China), and the sequencing results were analyzed based on amplicon sequence variants (ASVs). Alpha diversity indices (including Chao1, Observed_species, Shannon, and Simpson indices) were calculated to evaluate microbial species evenness. Beta diversity was evaluated by non-metric multidimensional scaling (NMDS) and partial least squares discriminant analysis (PLS-DA) based on the unweighted UniFrac distance. The linear discriminant analysis (LDA) combined effect size (LEfSe) measurements were used to identify the differences in microbial composition among the four groups.

### Statistical analysis

All data (excluding microbiota data) were analyzed as a 2 × 2 factorial arrangement with each piglet as the experimental unit by ANOVA using the general linear model procedure. Multiple comparisons of the means were conducted using the Tukey post-hoc test in the SPSS 22.0 (SPSS Inc., Chicago, IL, United States) when the *p*-value for the interaction was <0.05. The statistical model includes the effects of BA, IUGR, and their interaction. The differences in the relative abundances of jejunal and ileal microbiota were assessed using Wilcoxon rank tests. Spearman’s correlation analysis was conducted to evaluate the potential relationship between digestive organ indexes and microbial abundances in the jejunum and ileum.

## Results

### Effects of dietary BA on digestive organ index of weaned piglets with NBW and IUGR

As shown in Table 1, IUGR decreased (*p* < 0.05) the weight and length of jejunum and pancreas weight, whereas increased (*p* < 0.05) ileum index compared with the NBW piglets. Dietary BA supplementation decreased (*p* < 0.05) the weight, length, and index of the jejunum and the weight and index of the pancreas of weaned piglets regardless of IUGR. Furthermore, dietary BA supplementation increased (*p* < 0.05) the ileal length of NBW piglets. There were interactions (*p* < 0.05) between BA and IUGR in the jejunum weight and the weight, length, and index of the ileum.

**Table 1:** Effects of dietary BA supplementation on digestive organ indexes of weaned piglets with NBW and IUGR.

Items	NBW		IUGR		SEM	*p*-values		
	−BA	+BA	−BA	+BA		IUGR	BA	IUGR*BA
**Jejunum**								
Weight (g)	542.14^a^	381.00^b^	387.44^b^	345.45^b^	17.30	<0.01	<0.01	0.04
Length (cm)	636.50	539.83	536.00	511.00	16.16	0.04	0.04	0.24
Index (g/kg)	29.26	21.90	26.46	23.28	1.00	0.71	<0.01	0.27
**Ileum**								
Weight (g)	296.73^b^	421.41^a^	420.22^a^	385.45^a^	17.07	0.42	0.18	0.02
Length (cm)	348.92^c^	556.83^a^	488.25^b^	480.18^b^	17.73	0.28	<0.01	<0.01
Index (g/kg)	15.77^b^	23.91^a^	28.63^a^	26.89^a^	1.22	<0.01	0.12	0.02
**Pancreas**								
Weight (g)	43.36	37.38	35.49	29.66	1.29	<0.01	0.01	0.97
Index (g/kg)	2.32	2.15	2.37	1.99	0.07	0.61	0.02	0.34

Data are expressed as means with pooled SEM (*n* = 11–12). ^a,b,c^Means with different superscript letters in the same row are significantly different (*p* < 0.05). BA, bile acids; NBW, normal birth weight; IUGR, intrauterine growth retardation. Index, ratio of jejunal/ileal weight to body weight −BA, piglets received a basal diet; +BA, piglets received a basal diet with 400 mg/kg BA.

### Effects of dietary BA on small intestinal morphology of weaned piglets with NBW and IUGR

As shown in Table 2, IUGR decreased (*p* < 0.05) the jejunal VH/CD of weaned piglets compared with the NBW piglets. Moreover, dietary BA supplementation decreased (*p* < 0.05) the ileal VH of the NBW piglets compared with those without BA supplementation. Furthermore, there were interactions (*p* < 0.05) between BA and IUGR on ileal VH and VH/CD of weaned piglets.

**Table 2:** Effects of dietary BA supplementation on small intestinal morphology of weaned piglets with NBW and IUGR.

Items	NBW		IUGR		SEM	*p*-values		
	−BA	+BA	−BA	+BA		IUGR	BA	IUGR*BA
**Jejunum**								
VH (μm)	475.62	432.02	394.48	421.13	13.66	0.09	0.75	0.19
CD (μm)	248.42	219.17	242.63	264.51	8.13	0.23	0.82	0.12
VH/CD	1.83	2.01	1.65	1.62	0.08	0.03	0.55	0.38
**Ileum**								
VH (μm)	436.83^a^	381.97^b^	371.53^b^	405.68^ab^	10.44	0.30	0.60	0.03
CD (μm)	166.79	169.64	178.23	170.75	4.19	0.48	0.80	0.56
VH/CD	2.68^a^	2.27^a,b^	2.10^b^	2.39^a,b^	0.08	0.16	0.71	0.04

Data are expressed as means with pooled SEM (*n* = 11–12). ^a,b^Means with different superscript letters in the same row are significantly different (*p* < 0.05). BA, bile acids; NBW, normal birth weight; IUGR, intrauterine growth retardation. −BA, piglets received a basal diet; +BA, piglets received a basal diet with 400 mg/kg BA. VH, villus height; CD, crypt depth; VH/CD, the ratio of VH to CD.

### Effects of dietary BA on plasma biochemical parameters of weaned piglets with NBW and IUGR

As shown in Table 3, IUGR increased (*p* < 0.05) the plasma CHE and GLU concentrations of weaned piglets compared with the NBW piglets. Dietary BA supplementation increased (*p* < 0.05) the plasma ALB, TG, and TP concentrations, whereas decreased (*p* < 0.05) AST, CHE, LDH, and NH_3_ concentrations of weaned piglets regardless of IUGR. Furthermore, there was an interaction (*p* < 0.05) between BA and IUGR in plasma UN concentration.

**Table 3:** Effects of dietary BA supplementation on plasma biochemical parameters of weaned piglets with NBW and IUGR.

Items	NBW		IUGR		SEM	*p*-values		
	−BA	+BA	−BA	+BA		IUGR	BA	IUGR*BA
ALB (g/L)	33.98	35.87	31.06	34.56	0.69	0.12	0.05	0.54
ALP (U/L)	275.17	286.83	274.00	258.82	8.90	0.43	0.92	0.46
ALT (U/L)	73.33	63.90	69.29	58.46	2.54	0.35	0.05	0.89
AMS (U/mL)	2.22	2.39	2.51	2.31	0.13	0.54	0.92	0.31
AST (U/L)	59.67	53.75	68.25	54.45	2.09	0.24	0.02	0.32
CHE (U/L)	589.42	569.25	696.08	579.09	15.99	0.05	0.02	0.10
GLU (mmol/L)	5.44	4.95	5.83	5.73	0.13	0.02	0.22	0.43
HDL-C (mmol/L)	0.81	0.88	0.81	0.90	0.03	0.91	0.14	0.89
LDH (U/L)	615.75	502.92	563.64	477.27	21.72	0.28	0.01	0.71
LDL-C (mmol/L)	1.40	1.49	1.37	1.30	0.06	0.35	0.93	0.54
NH_3_ (μmol/L)	240.11	167.63	220.88	176.55	9.98	0.78	<0.01	0.45
TBA (mmol/L)	13.80	39.07	17.33	44.03	3.61	0.51	<0.01	0.91
TC (mmol/L)	2.36	2.50	2.28	2.45	0.06	0.57	0.18	0.87
TG (mmol/L)	0.41	0.65	0.44	0.88	0.05	0.09	<0.01	0.20
TP (g/L)	51.38	52.93	50.37	54.22	0.67	0.92	0.04	0.39
UN (mmol/L)	2.23^b^	2.96^a^	3.26^a^	2.57^b^	0.15	0.29	0.95	0.02

Data are expressed as means with pooled SEM (*n* = 11–12). ^a,b^Means with different superscript letters in the same row are significantly different (*p* < 0.05). BA, bile acids; NBW, normal birth weight; IUGR, intrauterine growth retardation. −BA, piglets received a basal diet; +BA, piglets received a basal diet with 400 mg/kg BA. ALB, albumin; ALP, alkaline phosphatase; ALT, alanine aminotransferase; AMS, amylase; AST, aspartate transaminase; CHE, choline esterase; GLU, glucose; HDL-C, high density lipoprotein-cholesterol; LDH, lactate dehydrogenase; LDL-C, low density lipoprotein-cholesterol; TC, total cholesterol; TG, triglyceride; TBA, total bile acids; TP, total protein; UN, urea nitrogen.

### Effects of dietary BA on plasma hormone concentrations of weaned piglets with NBW and IUGR

As shown in Table 4, IUGR increased (*p* < 0.05) the plasma Gas and LEP concentrations of weaned piglets compared with the NBW piglets. Dietary BA supplementation decreased (*p* < 0.05) the plasma Ghr concentration, and increased (*p* < 0.05) INS concentration of weaned piglets regardless of IUGR. However, there was no interaction (*p* > 0.05) between BA and IUGR in the plasma hormones of weaned piglets.

**Table 4:** Effects of dietary BA supplementation on plasma hormone concentrations of weaned piglets with NBW and IUGR.

Items	NBW		IUGR		SEM	*p*-values		
	−BA	+BA	−BA	+BA		IUGR	BA	IUGR*BA
CCK (ng/L)	701.29	791.64	726.73	766.98	18.62	0.08	0.99	0.50
Gas (pg/mL)	480.14	483.18	493.99	534.80	6.74	0.01	0.08	0.13
GC (pg mL)	38.07	40.28	36.82	44.34	2.02	0.73	0.24	0.52
Ghr (μg/L)	2.23	2.12	2.35	1.83	0.06	0.48	0.01	0.09
IGF-1 (μg/L)	14.59	13.74	13.37	14.15	0.21	0.33	0.94	0.05
IGF-2 (μg/L)	15.93	15.57	19.32	17.66	0.96	0.60	0.16	0.74
INS (mIU/L)	55.87	63.14	59.29	67.88	1.48	0.14	0.01	0.81
LEP (μg/L)	1.78	1.77	1.86	1.95	0.03	0.02	0.46	0.35
PP (ng/mL)	4.19	3.53	3.52	3.35	0.14	0.11	0.12	0.36
PYY (pg/mL)	287.04	323.42	312.07	361.51	12.04	0.19	0.08	0.78
SS (ng/L)	68.51	62.13	63.95	63.94	1.02	0.11	0.49	0.12

Data are expressed as means with pooled SEM (*n* = 11–12). BA, bile acids; NBW, normal birth weight; IUGR, intrauterine growth retardation. −BA, piglets received a basal diet; +BA, piglets received a basal diet with 400 mg/kg BA. CCK, cholecystokinin; Gas, gastrin; GC, glucocorticoid; Ghr, ghrelin; IGF-1, insulin-like growth factor 1; IGF-2, insulin-like growth factor 2; INS, insulin; LEP, leptin; PP, pancreatic polypeptide; PYY, peptide YY; SS, somatostatin.

### Effects of dietary BA on small intestinal digestive enzyme levels of weaned piglets with NBW and IUGR

As shown in Table 5, IUGR increased (*p* < 0.05) the trypsin level in the ileum of weaned piglets compared with the NBW piglets. Dietary BA supplementation decreased (*p* < 0.05) the trypsin and lipase levels in the jejunum and lipase in the ileum of weaned piglets regardless of IUGR. Furthermore, there was no interaction (*p* > 0.05) between BA and IUGR on the small intestinal enzyme level of weaned piglets.

**Table 5:** Effects of dietary BA supplementation on small intestinal digestive enzyme levels of weaned piglets with NBW and IUGR.

Items U/L prot	NBW		IUGR		SEM	*P*-values		
	−BA	+BA	-BA	+BA		IUGR	BA	IUGR*BA
**Jejunum**								
Amylase	23.21	24.94	28.82	23.91	1.08	0.12	0.80	0.96
Lipase	20.67	16.91	23.35	15.71	1.10	0.72	<0.01	0.35
Trypsin	32.94	25.29	30.77	23.80	1.53	0.54	0.02	0.91
**Ileum**								
Amylase	34.15	28.40	34.31	32.86	1.39	0.41	0.20	0.44
Lipase	18.96	15.06	18.71	14.79	0.74	0.85	<0.01	0.99
Trypsin	31.27	33.94	38.40	38.04	1.01	<0.01	0.54	0.42

Data are expressed as means with pooled SEM (*n* = 11–12). BA, bile acids; NBW, normal birth weight; IUGR, intrauterine growth retardation. −BA, piglets received a basal diet; +BA, piglets received a basal diet with 400 mg/kg BA.

### Effects of dietary BA on small intestinal microbial diversity of weaned piglets with NBW and IUGR

**Figure 1:**
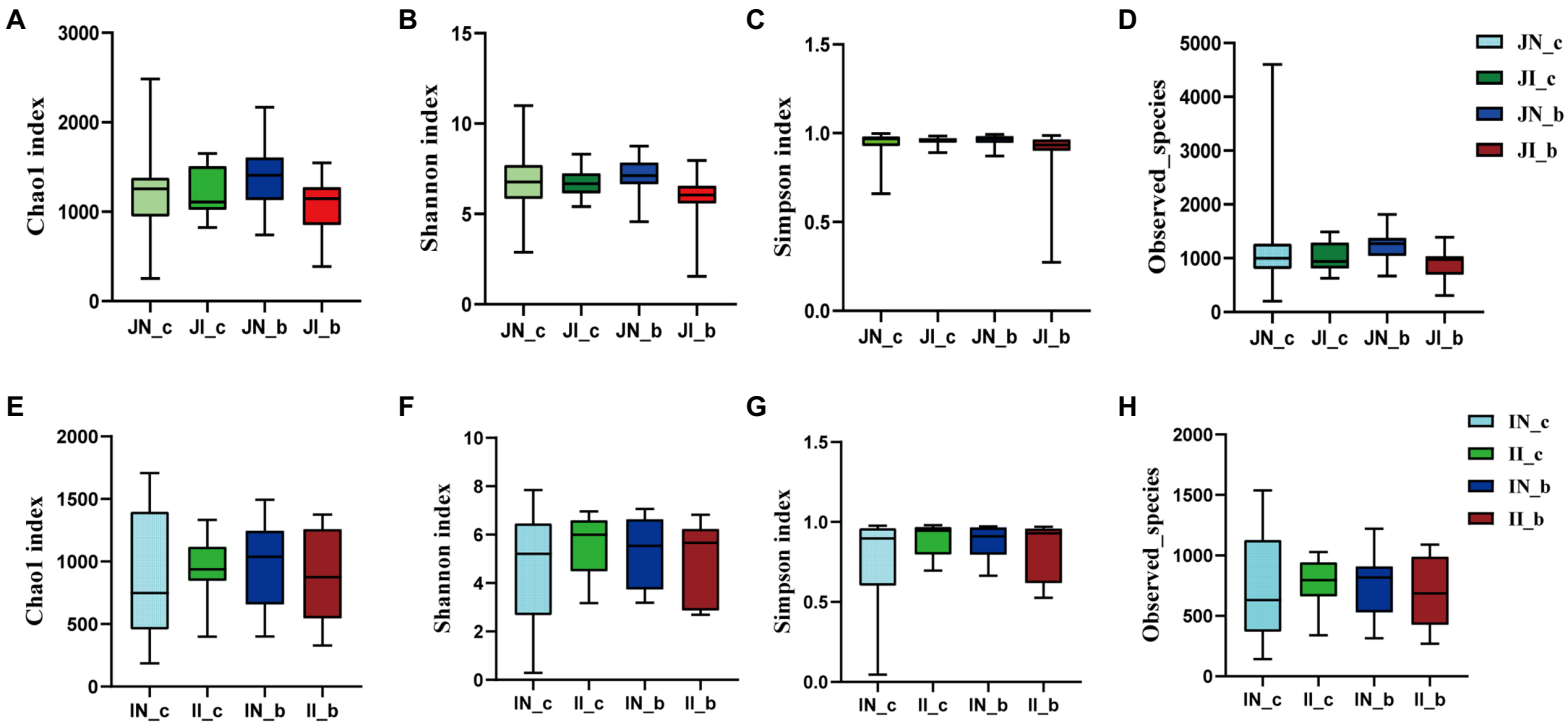
The alpha diversity of the jejunal **(A–D)** and ileal **(E–H)** microbial community estimated by Chao1 **(A,E)**, Shannon **(B,F)**, Simpson **(C,G)**, and Observed_species **(D,H)** indices in weaned piglets with NBW and IUGR (*n* = 11–12). BA, bile acids; NBW, normal birth weight; IUGR, intrauterine growth retardation. N_c, NBW piglets received a basal diet; N_b, NBW piglets received a basal diet with 400 mg/kg BA; I_c, IUGR piglets received a basal diet; and I_b, IUGR piglets received a basal diet with 400 mg/kg BA.

**Figure 2:**
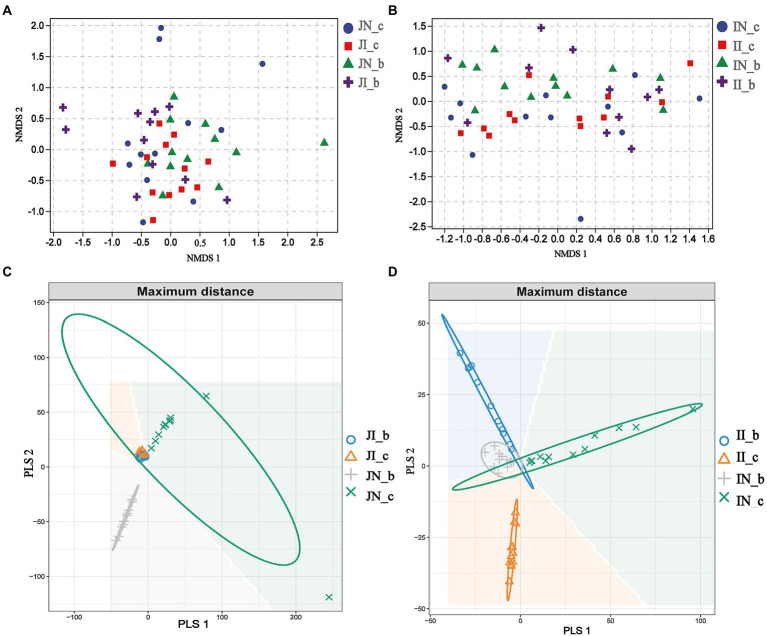
The nonmetric multidimensional scaling (NMDS) and partial least squares discriminant analysis (PLS-DA) of the jejunal (**[A,C]**, respectively) and ileal (**[B,D]**, respectively) microbial community of weaned piglets with NBW and IUGR (*n* = 11–12). Each symbol represents the gut microbiota of one piglet. BA, bile acids; NBW, normal birth weight; IUGR, intrauterine growth retardation. N_c, NBW piglets received a basal diet; N_b, NBW piglets received a basal diet with 400 mg/kg BA; I_c, IUGR piglets received a basal diet; and I_b, IUGR piglets received a basal diet with 400 mg/kg BA.

In the present study, a total of 3,845,457 and 3,143,008 high-quality 16S rRNA gene sequences were generated from jejunal and ileal samples of weaned piglets, respectively. The average numbers of high-quality sequences in the jejunum and ileum were 81,818 and 66,872 from the microbial community, respectively. The alpha diversity indices of the intestinal microbiota of four groups are shown in Figure 1. The Shannon, Simpson, Observed_species, and Chao 1 indices showed that there were no distinct separations in the jejunal and ileal microbiota among the four groups. The NMDS showed that the jejunal and ileal microbial community structure was relatively similar (Figures 2A,B). Furthermore, the PLS-DA indicated that the microbial community structure of the jejunum (Figure 2C) and ileum (Figure 2D) were clustered into four groups.

### Effects of dietary BA on small intestinal microbial community of weaned piglets with NBW and IUGR

**Figure 3:**
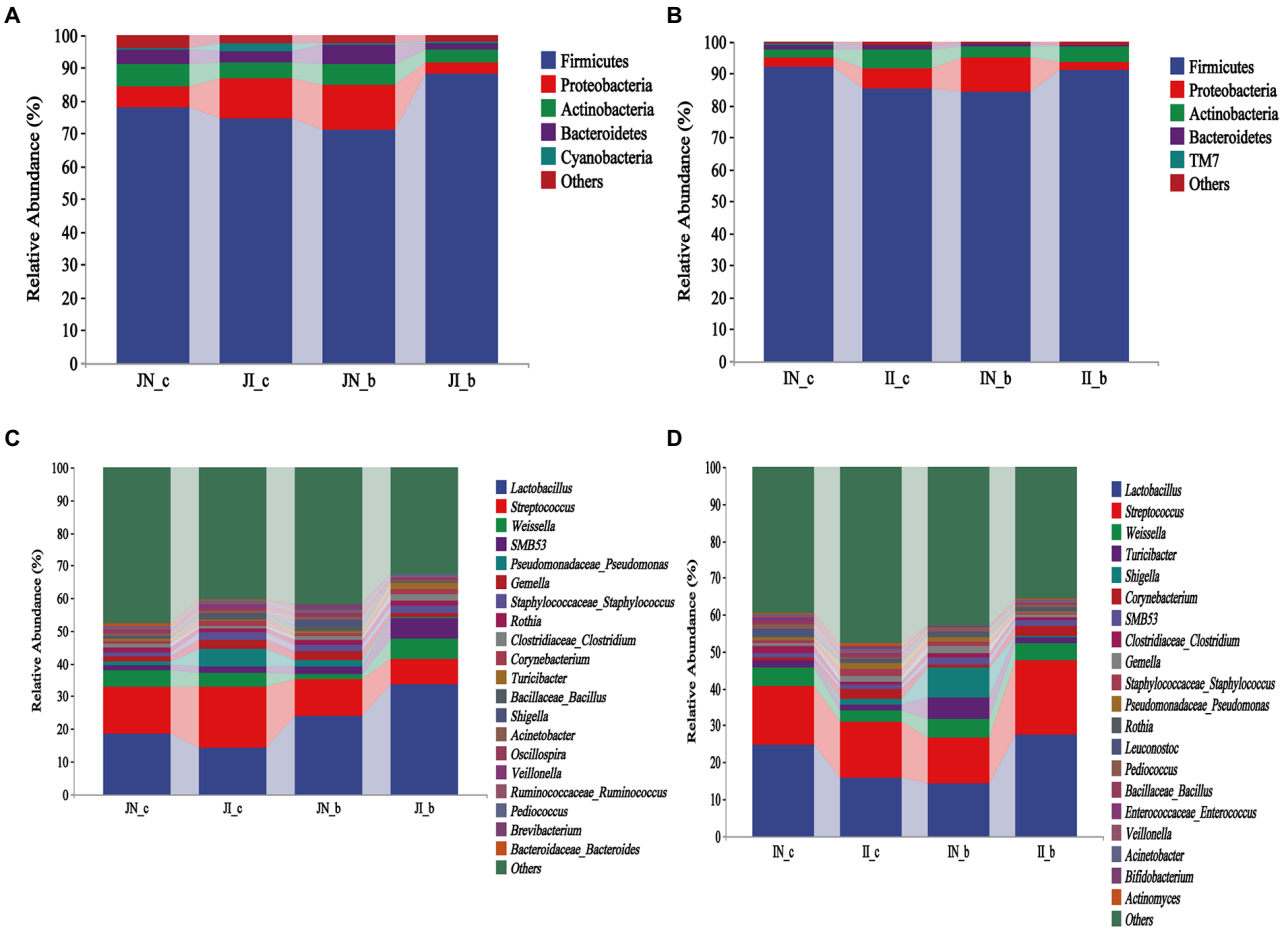
Effects of dietary BA supplementation on small intestinal microbial composition at the phylum **(A,B)** and genus **(C,D)** levels in weaned piglets with NBW and IUGR (*n* = 11–12). **(A,C)** Represent jejunal microbiota at the phylum **(A)** and genus **(C)** levels; **(B,D)** represent ileal microbiota at the phylum **(B)** and genus **(D)** levels. J, jejunum; I, ileum; N_c, NBW piglets received a basal diet; N_b, NBW piglets received a basal diet with 400 mg/kg BA; I_c, IUGR piglets received a basal diet; and I_b, IUGR piglets received a basal diet with 400 mg/kg BA.

As shown in Figure 3, Firmicutes, Proteobacteria, Actinobacteria, and Bacteroidetes were the most dominant phyla both in the jejunum and ileum of piglets in the four groups (Figures 3A,B). At the genus level, *Lactobacillus* and *Streptococcus* were the most dominant in the jejunum and ileum of weaned piglets among the four groups (Figures 3C,D). In the jejunum, *Lactobacillus* (18.34%), *Streptococcus* (14.71%), and *Weissella* (4.79%) were the most abundant in the NBW piglets and *Lactobacillus* (14.1%), *Streptococcus* (18.65%), and *Pseudomonas* (5.4%) in the IUGR piglets. In addition, *Lactobacillus* (23.82%), *Streptococcus* (11.18%), and *Gemella* (2.55%) were the most abundant in the NBW piglets with BA supplementation, and *Lactobacillus* (33.46%), *Streptococcus* (7.95%), and *Weissella* (6.22%) in the IUGR piglets with BA supplementation (Figure 3B). In the ileum, *Lactobacillus* (24.65%), *Streptococcus* (16.13%), and *Weissella* (4.81%) in the NBW piglets and *Lactobacillus* (15.74%), *Streptococcus* (15.11%), and *Weissella* (3.25%) in the IUGR piglets were the most abundant genera. In addition, *Lactobacillus* (14.07%), *Streptococcus* (12.39%), and *Shigella* (8.34%) were the most abundant in the NBW piglets with BA supplementation, whereas *Lactobacillus* (27.47%), *Streptococcus* (20.26%), and *Weissella* (4.58%) in the IUGR piglets with BA supplementation (Figure 3D).

**Figure 4:**
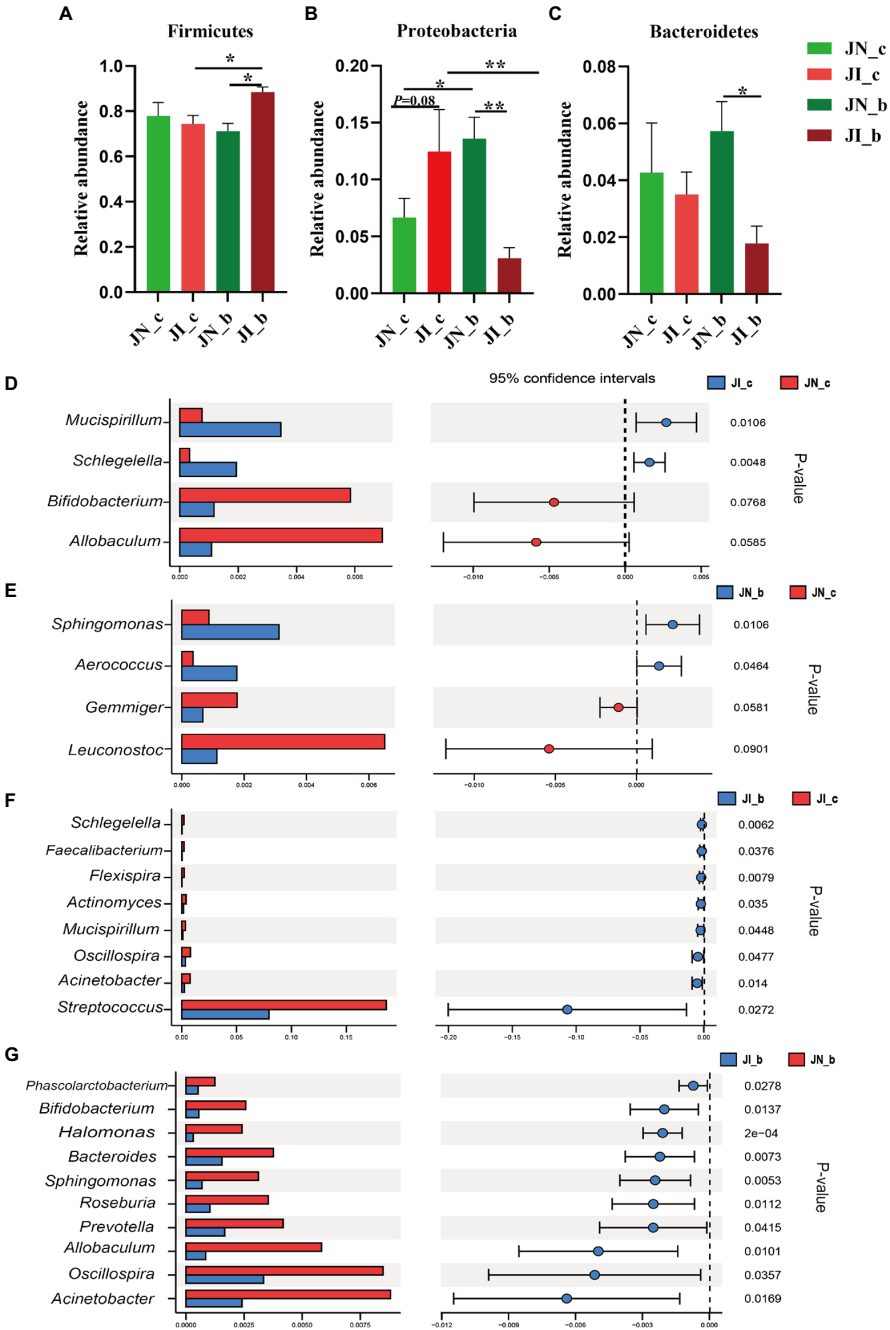
Effects of dietary BA supplementation on the differences in jejunal microbiota at the phylum **(A–C)** and genus **(D–G)** levels in weaned piglets with NBW and IUGR (*n* = 11–12). BA, bile acids; NBW, normal birth weight; IUGR, intrauterine growth retardation. J, jejunum; N_c, NBW piglets received a basal diet; N_b, NBW piglets received a basal diet with 400 mg/kg BA; I_c, IUGR piglets received a basal diet; and I_b, IUGR piglets received a basal diet with 400 mg/kg BA. D, JN_c vs. JI_c; E, JN_b vs. JN_c; F, JI_b vs. JI_c; G, JN_b vs. JI_b. ^*^p < 0.05, ^**^p < 0.01.

As shown in Figure 4, at the phylum level, dietary BA supplementation increased (*p* < 0.05) the jejunal Proteobacteria abundance of the NBW piglets, whereas it decreased (*p* < 0.05) in the IUGR piglets. Dietary BA supplementation to IUGR piglets increased (*p* < 0.05) the jejunal Firmicutes abundance, whereas decreased (*p* < 0.05) the jejunal Proteobacteria abundance compared with the IUGR piglets without BA supplementation. Moreover, dietary BA supplementation to IUGR piglets increased (*p* < 0.05) the jejunal Firmicutes abundance, whereas decreased (*p* < 0.05) the jejunal Proteobacteria and Bacteroidetes abundances compared to the NBW piglets with BA supplementation (Figures 4A–C).

At the genus level, IUGR increased (*p* < 0.05) the abundances of *Mucispirillum* and *Schlegelella* in the jejunum of piglets compared with the NBW piglets (Figure 4D); dietary BA supplementation to NBW piglets increased (*p* < 0.05) the jejunal *Sphingomonas* and *Aerococcus* abundances compared with the NBW piglets (Figure 4E). Dietary BA supplementation to IUGR piglets decreased (*p* < 0.05) the abundances of jejunal *Schlegelella*, *Faecalibacterium*, *Flexispira*, *Actinomyces*, *Mucisoirillum*, *Oscillospiea*, *Acinetobacter*, and *Streptococcus* compared with the IUGR piglets (Figure 4F). Moreover, dietary BA supplementation to IUGR piglets decreased (*p* < 0.05) the jejunal *Phascolarctobacterium*, *Bifidobacterium*, *Halomonas*, *Bacteroides*, *Sphingomonas*, *Roseburia*, *Prevotella*, *Allobaculum*, *Oscillospira*, and *Acinetobacter* abundances compared to the NBW piglets with BA supplementation (Figure 4G).

**Figure 5:**
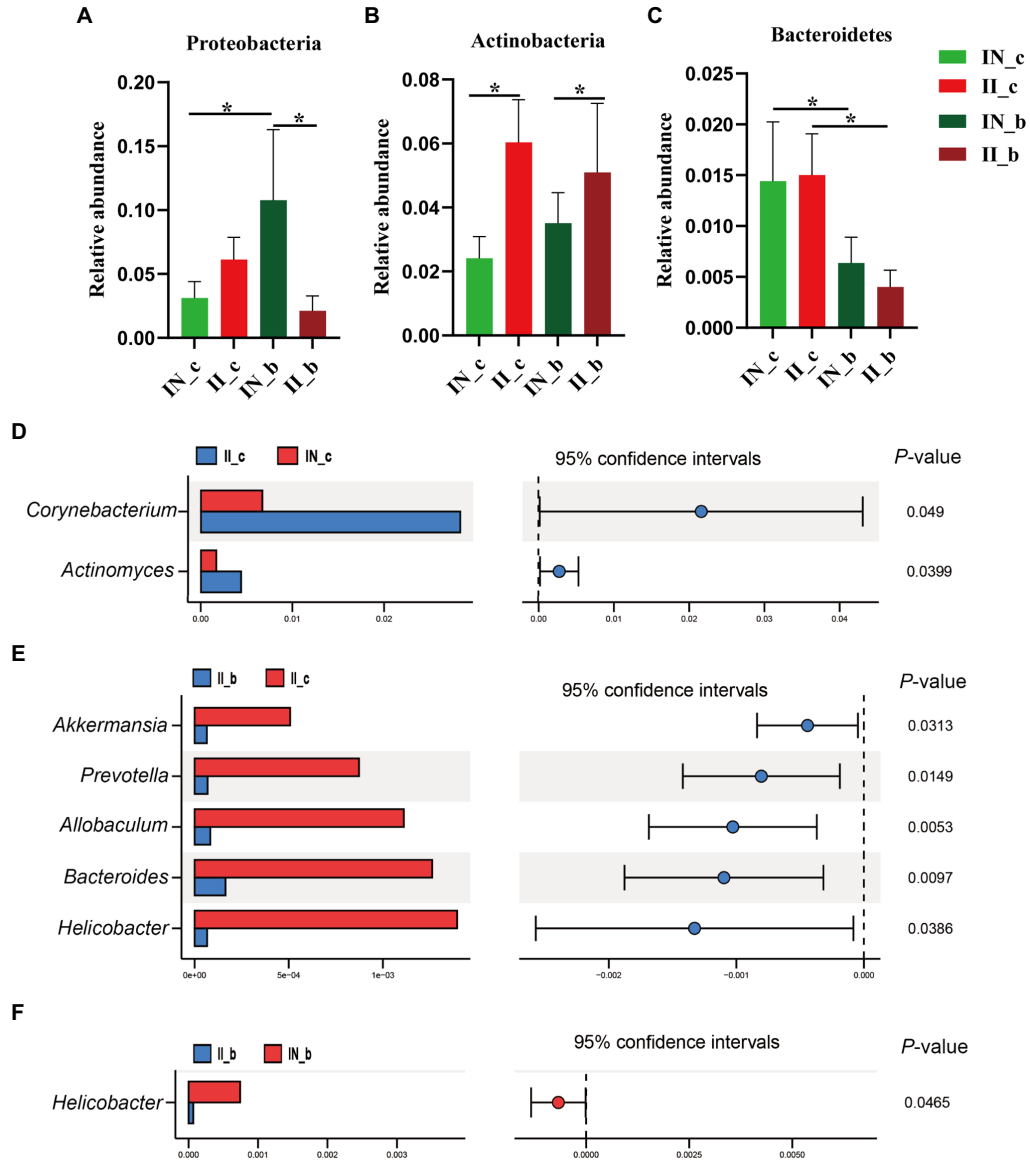
Effects of dietary BA supplementation on the differences in ileal microbiota at the phylum **(A–C)** and genus **(D–F)** levels in weaned piglets with NBW and IUGR (*n* = 11–12). BA, bile acids; NBW, normal birth weight; IUGR, intrauterine growth retardation. I, ileum; N_c; NBW piglets received a basal diet; N_b, NBW piglets received a basal diet with 400 mg/kg BA; I_c, IUGR piglets received a basal diet; I_b, IUGR piglets received a basal diet with 400 mg/kg BA. D, IN_c vs. II_c; E, II_b vs. II_c; F, IN_b vs. II_b. ^*^p < 0.05.

As shown in Figure 5, at the phylum level, IUGR increased (*p* < 0.05) the ileal Actionbacteria abundance of weaned piglets compared with the NBW piglets; dietary BA supplementation to NBW piglets increased (*p* < 0.05) the ileal abundance of Proteobacteria and decreased (*p* < 0.05) Bacteroidetes, whereas dietary BA supplementation to IUGR piglets decreased (*p* < 0.05) the ileal Bacteroidetes. Moreover, dietary BA supplementation to IUGR piglets decreased (*p* < 0.05) the ileal Proteobacteria abundance and increased (*p* < 0.05) ileal Actinobacteria compared to the NBW piglets with BA supplementation (Figures 5A–C). At the genus level, IUGR increased (*p* < 0.05) *Corynebacterium* and *Actinomyces* abundances (Figure 5D), and dietary BA supplementation to IUGR piglets decreased (*p* < 0.05) *Akkermansia*, *Prevotella*, *Allobaculum*, *Bacteroides*, and *Helicobacter* abundances in the ileum of weaned piglets (Figure 5E). Furthermore, dietary BA supplementation to IUGR piglets had a lower (*p* < 0.05) ileal *Helicobacter* abundance compared to the NBW piglets supplemented with BA (Figure 5F).

### Effects of dietary BA on intestinal microbial biomarkers of weaned piglets with NBW and IUGR

**Figure 6:**
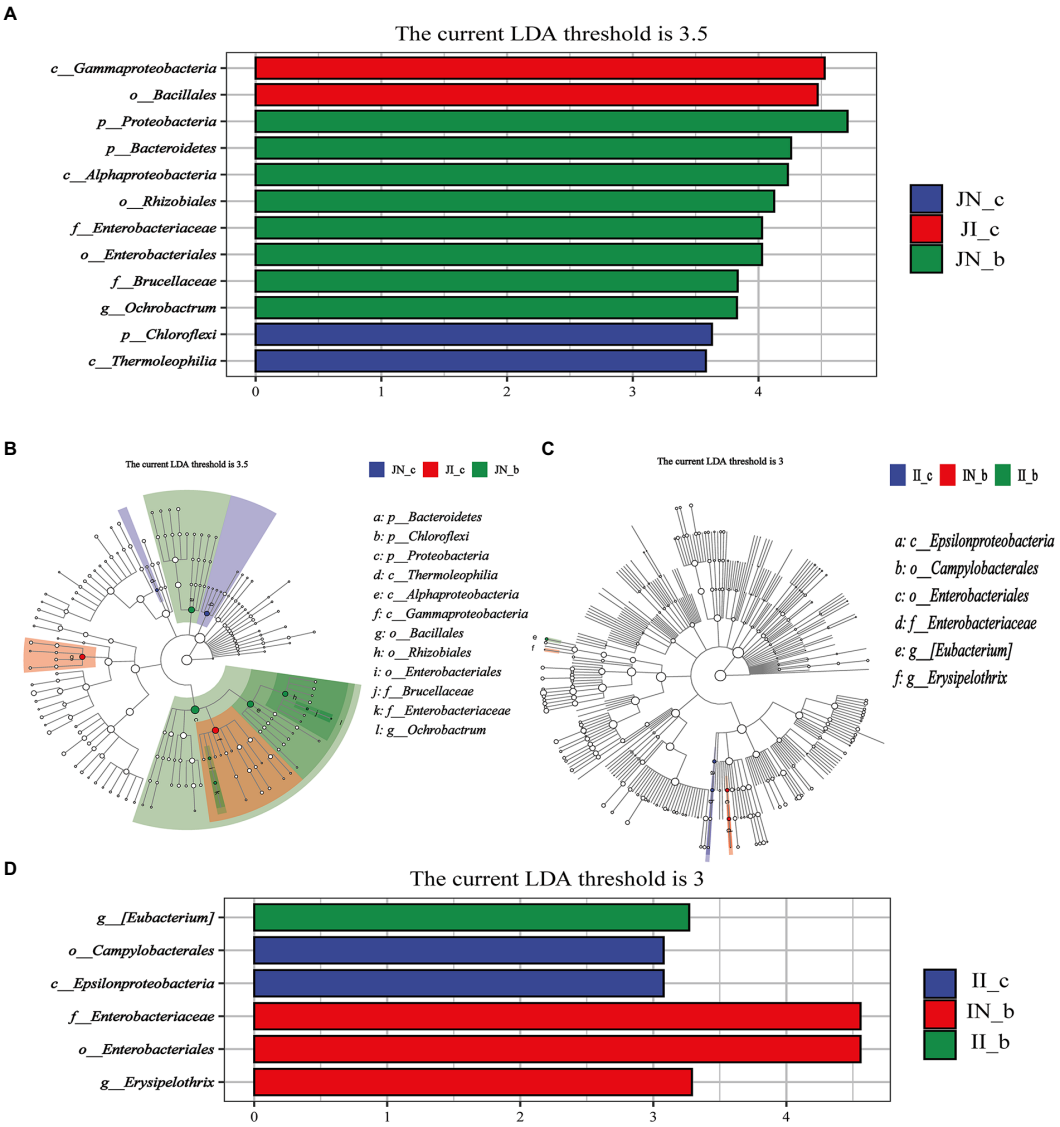
Linear discriminant analysis combined effect size measurements (LEfSe) analysis of the jejunal **(A,B)** and ileal **(C,D)** microbiota in weaned piglets with NBW and IUGR (*n* = 11–12). BA, bile acids; NBW, normal birth weight; IUGR, intrauterine growth retardation. J, jejunum; I, ileum; the green in the phylogenetic tree represents the microbial species that play an important role in the groups. The white nodes represent the species with no significant difference. **(A,B)** Represent the Linear discriminant analysis and Cladogram of jejunal microbiota, respectively; **(C,D)** Represent the Cladogram and Linear discriminant analysis of ileal microbiota, respectively. N_c; NBW piglets received a basal diet; N_b, NBW piglets received a basal diet with 400 mg/kg BA; I_c, IUGR piglets received a basal diet; I_b, IUGR piglets received a basal diet with 400 mg/kg BA.

The jejunal microbial biomarkers are shown in Figures 6A,B. The LEfSe analysis results (LDA >3.5, *p* < 0.05) showed that the microbial biomarkers of *Gammaproteobacteria* and *Bacillales* were detected in the IUGR piglets, and *Chloroflexi* and *Thermoleophilia* were detected in the NBW piglets. Moreover, the microbial biomarkers of *Proteobacteria*, *Bacteroidetes*, *Alphaproteobacteria*, *Rhizobiales*, *Enterobacteriaceae*, *Enterobacteriales*, *Brucellaceae*, and *Ochrobactrum* were detected in the NBW piglets with BA supplementation.

The ileal microbial biomarkers are shown in Figure 6C,D. The microbial biomarkers (LDA > 3, *p* < 0.05) of *Campylobacterales* and *Epsilonproteobacteria* were detected in the IUGR piglets. Dietary BA supplementation to NBW piglets enriched three microbial biomarkers, including *Enterobacteriaceae*, *Enterobacteriales*, and *Erysipelothrix*, whereas dietary BA supplementation to IUGR piglets enriched the [*Eubacterium*].

### Effects of dietary BA on metabolic profiles of small intestinal microbiota of weaned piglets with NBW and IUGR

**Figure 7:**
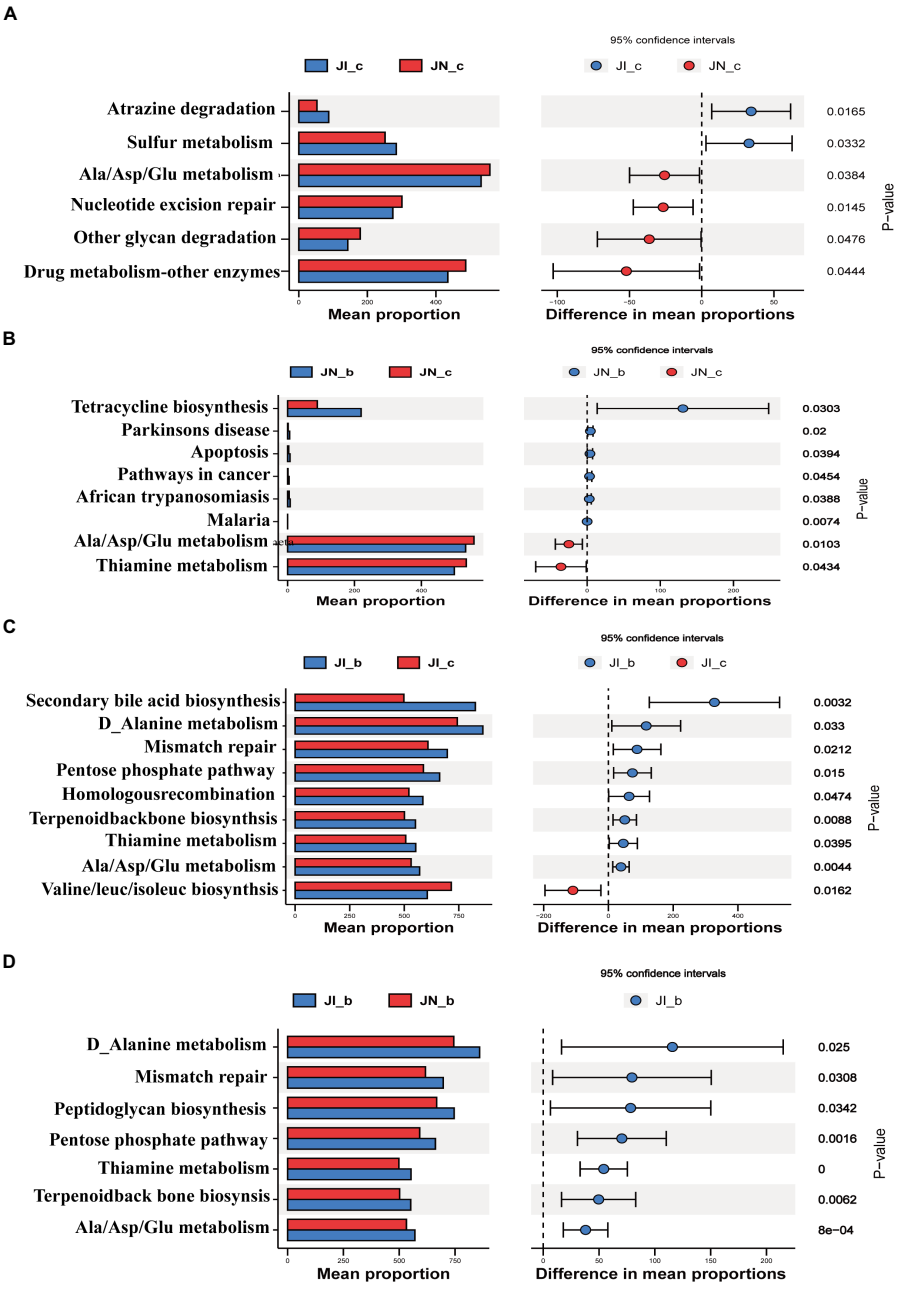
Functional prediction by Phylogenetic Investigation of Communities by Reconstruction of Unobserved States (PICRUSt) of the jejunal microbiota in weaned pigs with NBW and IUGR (*n* = 11–12). (A) JI_c vs. JN_c, (B) JN_b vs. JN_c, (C) JI_b vs. JI_c, and (D) JI_b vs. JN_b. BA, bile acids; NBW, normal birth weight; IUGR, intrauterine growth retardation. J, jejunum; A, JN_c vs. JI_c; B, JN_c vs. JN_b; C, JI_c vs. JI_b; D, JI_b vs. JI_b. N_c, NBW piglets received a basal diet; N_b, NBW piglets received a basal diet with 400 mg/kg BA; I_c, IUGR piglets received a basal diet; I_b, IUGR piglets received a basal diet with 400 mg/kg BA.

The PICRUSt2 was used to predict the metabolic ability of microbial communities (Figure 7). The relative abundances of jejunal microbial genes involved in atrazine degradation and sulfur metabolism were increased (*p* < 0.05), and the microbial genes involved in alanine/aspartate/glutamate metabolism, nucleotide excision repair, other glycan degradation, and drug metabolism were decreased (*p* < 0.05) in the IUGR piglets compared to the NBW piglets. Dietary BA supplementation to NBW piglets increased (*p* < 0.05) the relative abundances of jejunal microbial genes involved in tetracycline biosynthesis, Parkinson’s disease, apoptosis, and pathways in cancer, while decreased (*p* < 0.05) malaria, alanine/aspartate/glutamate metabolism, and thiamine metabolism compared to the NBW piglets without BA supplementation.

In addition, dietary BA supplementation to the IUGR piglets decreased (*p* < 0.05) the relative abundances of jejunal microbial genes involved in secondary BA biosynthesis, D-alanine metabolism, mismatch repair, pentose phosphate pathway, homologous recombination, terpenoid backbone biosynthesis, thiamine metabolism, and alanine/aspartate/glutamate metabolism, while increased (*p* < 0.05) valine/leucine/isoleucine biosynthesis and C5-branched dibasic acid metabolism compared to the IUGR piglets without BA supplementation. Furthermore, dietary BA supplementation to IUGR piglets increased (*p* < 0.05) the relative abundances of jejunal microbial genes involved in D-alanine metabolism, mismatch repair, peptidoglycan biosynthesis, pentose phosphate pathway, thiamine metabolism, terpenoid backbone biosynthesis, and alanine/aspartate/glutamate metabolism compared to the NBW piglets with BA supplementation.

**Figure 8:**
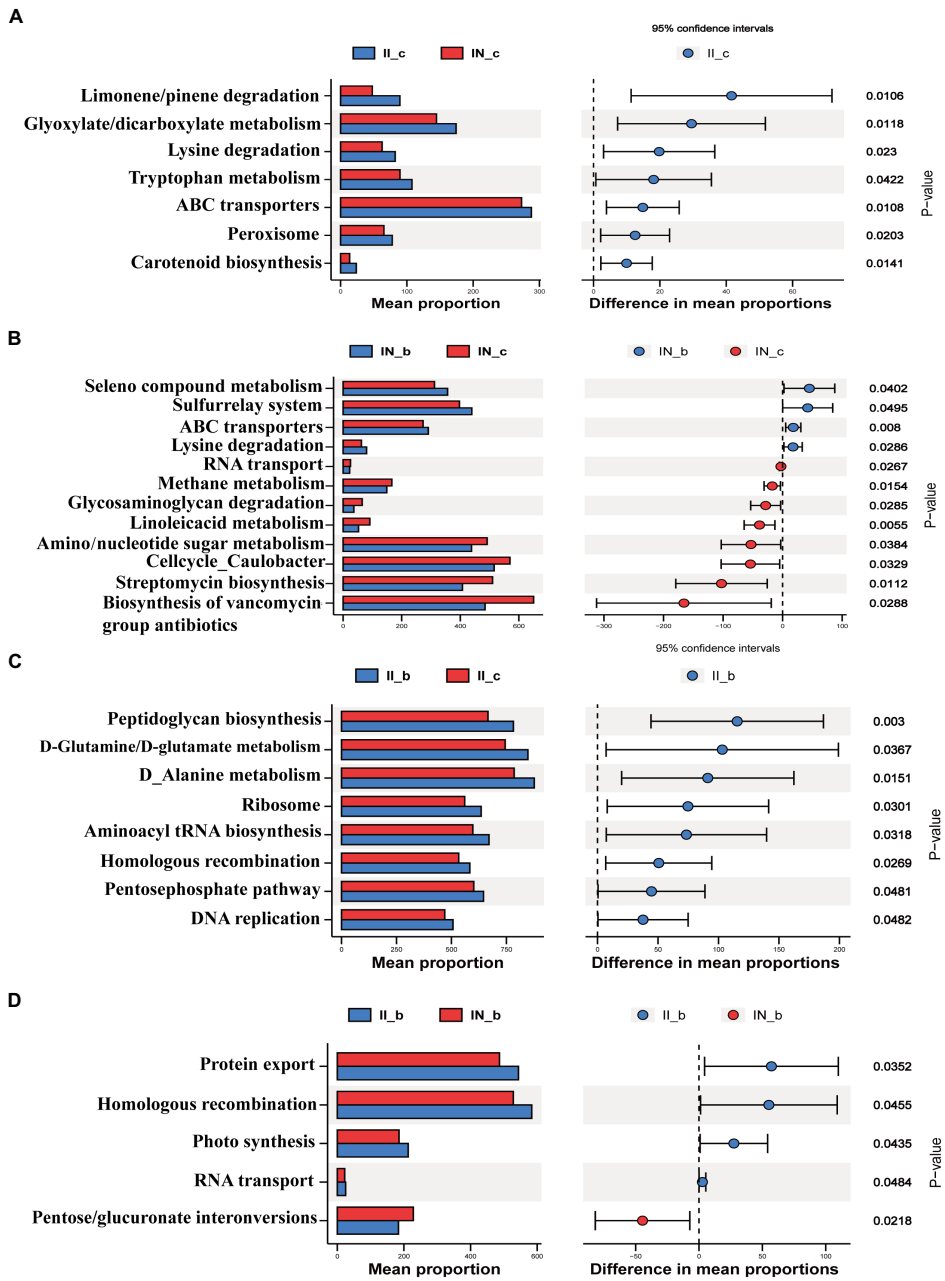
Functional prediction by Phylogenetic Investigation of Communities by Reconstruction of Unobserved States (PICRUSt) of the ileal microbiota in weaned piglets with NBW and IUGR (*n* = 11–12). (A) II_c vs. IN_c, (B) IN_b vs. IN_c, (C) II_b vs. II_c, and (D) II_b vs. IN_b. BA, bile acids; NBW, normal birth weight; IUGR, intrauterine growth retardation. I, ileum; A, IN_c vs. II_c; B, IN_c vs. IN_b; C, II_c vs. II_b; D, IN_b vs. II_b. N_c, NBW piglets received a basal diet; N_b, NBW piglets received a basal diet with 400 mg/kg BA; I_c, IUGR piglets received a basal diet; I_b, IUGR piglets received a basal diet with 400 mg/kg BA.

As shown in Figure 8, IUGR increased (*p* < 0.05) the relative abundances of the ileal microbial genes involved in carotenoid biosynthesis, peroxisome, ABC transporters, tryptophan metabolism, lysine degradation, glyoxylate and dicarboxylate metabolism, and limonene and pinene degradation compared to the NBW piglets. Dietary BA supplementation to NBW piglets increased (*p* < 0.05) the relative abundances of the ileal microbial genes involved in selenocompound metabolism, sulfurrelay system and ABC transporters, and lysine degradation, while decreased (*p* < 0.05) RNA transport, methane metabolism, glycosaminoglycan degradation, linoleic acid metabolism, amino sugar/nucleotide sugar metabolism, cell cycle caulobacter, streptomycin biosynthesis, and biosynthesis of vancomycin group antibiotics compared to the NBW piglets without BA supplementation. Dietary BA supplementation to IUGR piglets increased (*p* < 0.05) the abundances of ileal microbial genes involved in peptidoglycan biosynthesis, D-glutamine and D-glutamate metabolism, D-alanine metabolism, ribosome, aminoacyl-tRNA biosynthesis, photosynthesis, homologous recombination, pentose phosphate pathway, and DNA replication compared to the IUGR piglets without BA supplementation. Moreover, dietary BA supplementation to IUGR piglets increased (*p* < 0.05) the relative abundances of ileal microbial genes involved in protein export, homologous recombination, photosynthesis, and RNA transport, whereas decreased (*p* < 0.05) pentose and glucuronate interconversions compared to the NBW piglets with BA supplementation.

### Correlation between intestinal microbial abundance and plasma hormones, biochemical parameters, and intestinal digestive enzyme levels of weaned piglets with NBW and IUGR

**Figure 9:**
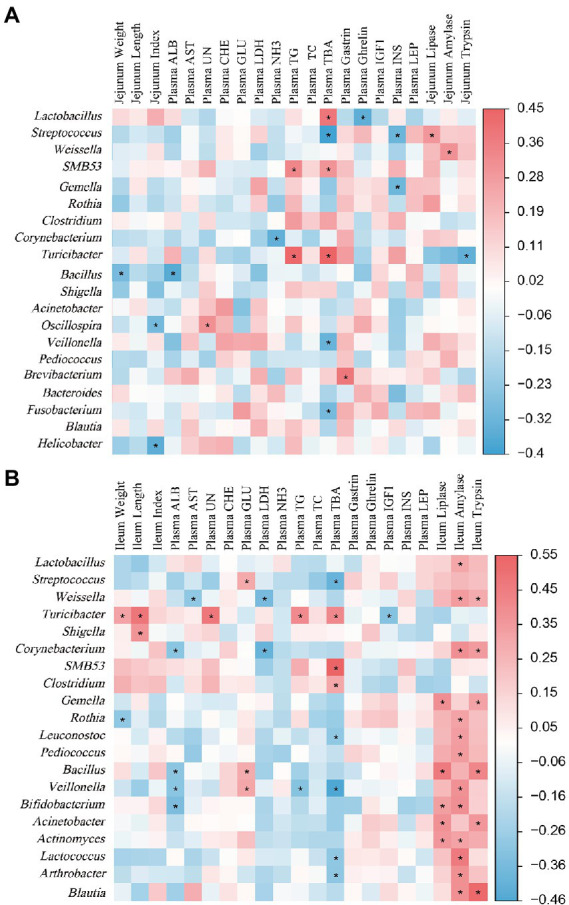
The Spearman’s correlation analysis between plasma parameters, small intestine-related indicators, and small intestinal microbiota. **(A)** Represents the correlation of jejunal microbiota and plasma parameters, jejunum-related indicators. **(B)** Represents the correlation of ileal microbiota and plasma parameters, ileum-related indicators. The red and blue represent a significantly positive correlation and negative correlation, respectively. ^*^p < 0.05.

The Spearman’s correlation analysis was performed to determine the relationship between jejunal microbial abundance and plasma hormones, biochemical parameters, intestinal indexes, and digestive enzyme levels (Figure 9A). The correlation analysis showed that the positive correlation (*p* < 0.05) included between *Lactobacillus* with plasma TBA concentration; *Streptococcus* with jejunal Lipase level; *Weissella* with jejunal amylase level; *SMB53* with plasma TG and TBA concentrations; *Turicibacter* with plasma TG and TBA concentrations; *Oscillospira* with plasma UN concentration; and *Brevibacterium* with plasma Gas concentration. In addition, the negative correlation (*p* < 0.05) included between *Lactobacillus* with plasma Ghr concentration; *Gemella* with plasma insulin concentration; *Veillonella* and *Fusobacterium* with plasma TBA concentration; *Corynebacterium* with plasma NH_3_ concentration; and Helicobacter with jejunal index.

As shown in Figure 9B, Spearman correlation analysis was performed to determine the relationship between ileal microbial abundance and plasma hormones, biochemical parameters, intestinal indexes, and digestive enzyme levels. *Lactobacillus* was positively (*p* < 0.05) correlated with ileal amylase level. *Streptococcus* was negatively (*p* < 0.05) correlated with plasma TBA concentration. *Weissella* was positively (*p* < 0.05) correlated with ileal amylase and trypsin levels, and was negatively (*p* < 0.05) correlated with plasma AST and LDH levels. *Turicibacter* was positively (*p* < 0.05) correlated with ileal length and plasma UN, TG, and TC concentrations, while was negatively (*p* < 0.05) correlated with plasma IGF-1 concentration. *Corynebacterium* was positively (*p* < 0.05) correlated with ileal amylase and trypsin levels, while was negatively (*p* < 0.05) correlated with final body weight and plasma ALB concentration. *Rothia* was positively (*p* < 0.05) correlated with ileal amylase level, while was negatively (*p* < 0.05) correlated with ileal weight. *Leuconostoc* was positively (*p* < 0.05) correlated with ileal amylase level, while was negatively (*p* < 0.05) correlated with plasma TBA concentration. *Veillonella* was positively (*p* < 0.05) correlated with ileal amylase level and plasma GLU concentration, while was negatively (*p* < 0.05) correlated with plasma ALB and TBA concentrations. *Bifidobacterium* was positively (*p* < 0.05) correlated with ileal lipase and amylase levels, while was negatively (*p* < 0.05) correlated with plasma ALB concentration. *Lactococcus* and *Arthrobacter* were positively (*p* < 0.05) correlated with ileal amylase level, while were negatively (*p* < 0.05) correlated with plasma TBA concentration. *SMB53* and *Clostridium* were positively (*p* < 0.05) correlated with plasma TBA concentration. *Gemella* and *Bacillus* were positively (*p* < 0.05) correlated with ileal lipase and trypsin levels. *Pediococcus* was positively (*p* < 0.05) correlated with ileal amylase level. *Acinetobacter* and *Blautia* were positively (*p* < 0.05) correlated with ileal amylase and trypsin levels.

## Discussion

The poor intestinal function and growth performance of IUGR piglets have been widely confirmed (Tao et al., 2019). Many strategies have been adopted to change this situation. The BA play an important role in promoting nutrient digestion and growth performance of animals (Lai et al., 2018). However, dietary BA supplementation had no impact on the growth performance of weaning piglets (data not shown) in the present study, which may be associated with the dosage and times of BA supplementation in the diet, and further studies are needed to explore the exact reason. In addition, IUGR impaired the small intestinal morphology, function, and microbial community, whereas dietary BA supplementation increased the ileum development in NBW piglets, and protected the liver function of weaned piglets. Furthermore, dietary BA can regulate the intestinal microbial community by promoting the proliferation of potentially beneficial bacteria.

The small intestine is an important digestive and absorptive organ (Wang et al., 2020), and its length is significantly associated with the growth traits of various farm animals, including pigs (Gao et al., 2010). Several studies reported that intestinal length and weight are proportional to the ratio of body weight gain of pigs (Meyer et al., 2014). Moreover, weaning is the critical period of intestinal development that affects the growth performance later in the life of pigs (Metzler-Zebeli et al., 2018). In the present study, IUGR decreased the jejunal weight and length, ileal index, and pancreas weight of weaned piglets, while dietary BA supplementation increased the ileal weight, length, and index of NBW piglets. A previous study showed that the higher ileal digestive characteristics displayed a higher absorption efficiency of nutrients (including amino acids) in the intestine (Zhu et al., 2018). These results suggest that dietary BA contributed to the intestinal development and maturation of weaned piglets regardless of IUGR, and further studies are needed to confirm the exact mechanism.

The morphological structure of the small intestine provides an indication of enterocyte functional capabilities and degrees of maturation, which determines the intestinal health and growth performance of animals (Ding et al., 2021). The higher VH showed an increased surface area, allowing for higher absorption of available nutrients and thus resulting in faster growth (He et al., 2011). Previous studies reported that the small intestinal villi of IUGR piglets were shorter and fewer than the NBW piglets (Olszewski et al., 2021). In the present study, IUGR decreased the VH/CD, which was consistent with a previous study (Dong et al., 2016). These findings suggest that IUGR can impair the small intestinal morphology of weaned piglets. However, dietary BA supplementation did not improve the small intestinal morphological structure of weaned piglets.

Plasma levels of TP and ALB reflect the absorption and metabolism of protein in pigs (Verheyen et al., 2007). Furthermore, plasma UN concentration is the indicator of protein and amino acid catabolism, and a decrease in the plasma UN level reflects that the amino acid metabolism is well balanced (Ma et al., 2020). In the present study, IUGR increased the plasma UN concentration of piglets, which was consistent with a previous study (Huang et al., 2020). Dietary BA supplementation increased plasma ALB and decreased plasma UN, CHE, and NH_3_ concentrations of weaned piglets. These results indicate a beneficial effect on the protein metabolism of weaned piglets.

The plasma TG, TC, HDL-C, and LDL-C concentrations can reflect the lipid utilization and absorption (Kong et al., 2016), and HDL-C can transport TC to the liver to metabolize other substances, such as BA, and maintain the TC balance of an individual (Tian et al., 2011). Previous studies found that IUGR presented lower plasma GLU and TG and higher plasma TC and LDL-C concentrations of weaned piglets (Huang et al., 2020). In the present study, IUGR did not change plasma TC, glucose, and LDL-C concentrations, while dietary BA supplementation increased plasma TG and TBA concentrations of weaned piglets, which may be postulated that BA can promote the digestion and absorption of fat from diets. Therefore, these findings suggest that dietary BA supplementation can facilitate the lipid metabolism of NBW and IUGR piglets.

Moreover, plasma AST and ALT levels are commonly used to evaluate the status of the liver function of an individual, and elevated enzyme level indicates the impaired liver function (Cheng et al., 2020). Previous studies showed that IUGR could increase the plasma AST and ALT levels of weaned piglets (Zhao et al., 2020), suggesting the impaired membrane of hepatocytes in piglets (Gao et al., 2020). In the present study, dietary BA supplementation decreased the plasma AST and ALT levels in both NBW and IUGR piglets. Collectively, these results showed that dietary BA supplementation had significant protective effects on the liver of NBW and IUGR piglets.

IUGR piglets are often experienced insufficient gastric acid secretion and low digestive enzyme level in the intestinal tract due to the immature digestive system, which results in the decreased digestion and absorption ability of nutrients of weaned piglets. BA have been found to improve the digestive enzyme level and promote the digestion and absorption of fats in food (McGlone and Bloom, 2019). Previous studies reported that dietary BA supplementation could increase intestinal trypsin level (Lammasak et al., 2019). Our results showed that IUGR increased ileal trypsin level of weaned piglets, while dietary BA supplementation decreased jejunal and ileal lipase levels in both NBW and IUGR piglets. Whereas a previous study also indicated that dietary BA supplementation effectively enhanced the levels of intestinal lipase and lipoprotein lipase in poultry (Lai et al., 2018). These findings suggest that the digestive level of the small intestine may be associated with dietary BA level.

The intestinal microbiota plays a critical role in nutrient digestion and absorption and host health (Martinez-Guryn et al., 2018) by maintaining the balance of intestinal microecology in animals (Wang et al., 2021). Firmicutes are associated with energy absorption from nutrients, while members of the Bacteroidetes are specialized in the degradation of proteins and carbohydrates (Zhang et al., 2019). Previous studies showed that phylum Actinobacteria, including *Bifidobacterium* is the key player in maintaining the gut barrier homeostasis (Hu et al., 2018). In the present study, dietary BA supplementation increased the jejunal Firmicutes and ileal Proteobacteria and Actinobacteria, whereas decreased the jejunal Proteobacteria and Bacteroidetes and ileal Bacteroidetes of IUGR piglets. In addition, *Lactobacillus* was the most dominant genera in jejunum and ileum of weaned piglets with BA supplementation. These results indicate that dietary BA supplementation significantly altered the composition of the small intestinal microbiota and increased the proportion of *Lactobacillus*, which might be associated with the small intestinal digestive enzyme level and could maintain the intestinal microbiota balance of weaned piglets.

Gut microbiota participates in the metabolism of host nutrients and produces various bioactive metabolites, which can be absorbed into the blood to maintain the normal physiological levels of an individual (Sun and Shen, 2018). However, these metabolites are also associated with several pathological phenotypes (Papizadeh et al., 2017). Previously, it has been reported that IUGR piglets had a lower abundance of microbial pathways related to carbohydrate, lipid, and amino acid metabolism and biosynthesis of other secondary metabolites (Xiong et al., 2020). Our results showed that dietary BA supplementation increased the abundance of microbial genes related to thiamine and alanine/aspartate/glutamate metabolism and the secondary BA biosynthesis in the small intestine of weaned piglets with NBW and IUGR. Moreover, dietary BA supplementation increased the abundance of microbial genes related to homologous recombination and pentose phosphate pathways in the small intestine of IUGR piglets. These results indicate that dietary BA supplementation could improve the small intestinal microbial metabolism, including amino acid, secondary BA, and glucose metabolism.

The gut microbiota can influence animal physiology by producing secondary BA. These secondary BA can act as signalling molecules to modulate host lipid, glucose, and energy metabolism and affect the intestinal microbiota community (Lucas et al., 2021). The deconjugation and absorption of BA occur mainly in the jejunum and ileum. In the present study, Spearman’s correlation analysis revealed that plasma TBA concentration was positively correlated with the jejunal *Lactobacillus* while negatively correlated with the jejunal *Streptococcus* and ileal *Veillonella*. Moreover, ileal *Veillonella* abundance was negatively correlated with plasma TG concentration while positively correlated with plasma GLU concentration. These results suggest that the jejunal and ileal microbiota can regulate the plasma BA, TG, and GLU concentrations, which might be associated with the regulation of BA in the glucolipid metabolism of piglets.

In addition, intestinal digestive enzyme level is associated with intestinal microbiota (Su and Yao, 2022). The present study observed a positive correlation between the ileal amylase and the abundances of ileal *Lactobacillus, Veillonella, Bifidobacterium,* and *Blautia*; and the ileal trypsin level with the abundances of ileal *Gemella*, *Bacillus*, and *Blautia*. These findings suggest that dietary BA could regulate host digestive capacity by regulating gut microbial community and digestive enzyme level.

## Conclusion

In summary, IUGR impaired the small intestinal function and microbial community, whereas dietary BA supplementation showed potential effects on the small intestinal function, protective liver, and regulated intestinal microecology by increasing the proportion of potentially beneficial bacteria in the small intestine of NBW and IUGR piglets. Collectively, BA could be effective additives in swine production.

## Data availability statement

The data presented in the study are deposited in the online repository, accession number can be found at https://www.ncbi.nlm.nih.gov/sra/PRJNA872301.

## Ethics statement

The animal study was reviewed and approved by the Animal Care and Use Committee of the Institute of Subtropical Agriculture, Chinese Academy of Sciences, Changsha, Hunan, China.

## Author contributions

XK and ZY: designed the experiments. YL and QZ: conducted the experiments. YL, MA, and XK: wrote the manuscript. XK, MA, ZY, and YL: revised the manuscript. All authors contributed to the article and approved the submitted version.

## Funding

This study was jointly supported by the Key Project of Regional Innovation and Development Joint Fund of National Natural Science Foundation of China (U20A2056), China Agriculture Research System of MOF and MARA (CARS-35), and the Special Funds for Construction of Innovative Provinces in Hunan Province (2019RS3022).

## Conflict of interest

The authors declare that the research was conducted in the absence of any commercial or financial relationships that could be construed as a potential conflict of interest.

## Publisher’s note

All claims expressed in this article are solely those of the authors and do not necessarily represent those of their affiliated organizations, or those of the publisher, the editors and the reviewers. Any product that may be evaluated in this article, or claim that may be made by its manufacturer, is not guaranteed or endorsed by the publisher.

## References

[ref400] Cai J. Sun L. Gonzalez F. J. (2020). Gut microbiota-derived bile acids in intestinal immunity, inflammation, and tumorigenesis. Cell Host Microbe. 30, 289–300. doi: 10.1016/j.chom.2022.02.004 PMC8923532 35271802

[ref1] Cheng K. Ji S. Jia P. Zhang H. Wang T. Song Z.. (2020). Resveratrol improves hepatic redox status and lipid balance of neonates with intrauterine growth retardation in a piglet model. Biomed. Res. Int. 2020:7402645. doi: 10.1155/2020/7402645, PMID: 32733952 PMC7383311

[ref2] Ding H. Zhao X. C. Azad M. A. K. Ma C. Gao Q. K. He J. H.. (2021). Dietary supplementation with *Bacillus subtilis* and xylo-oligosaccharides improves growth performance and intestinal morphology and alters intestinal microbiota and metabolites in weaned piglets. Food Funct. 12, 5837–5849. doi: 10.1039/d1fo00208b, PMID: 34018533

[ref3] Dong L. Zhong X. He J. Zhang L. Bai K. Xu W.. (2016). Supplementation of tributyrin improves the growth and intestinal digestive and barrier functions in intrauterine growth-restricted piglets. Clin. Nutr. 35, 399–407. doi: 10.1016/j.clnu.2015.03.002, PMID: 26112894

[ref4] Ferenc K. Pietrzak P. Godlewski M. M. Piwowarski J. Kiliańczyk R. Guilloteau P.. (2014). Intrauterine growth retarded piglet as a model for humans-studies on the perinatal development of the gut structure and function. Reprod. Biol. 14, 51–60. doi: 10.1016/j.repbio.2014.01.005, PMID: 24607255

[ref5] Gao J. Ren J. Zhou L. H. Ren D. R. Li L. Xiao S. J.. (2010). A genome scan for quantitative trait loci affecting the length of small intestine in a white Duroc × Chinese Erhualian intercross resource population. J. Anim. Breed. Genet. 127, 119–124. doi: 10.1111/j.1439-0388.2009.00816.x, PMID: 20433520

[ref6] Gao H. Zhang L. Wang L. Liu X. Hou X. Zhao F.. (2020). Liver transcriptome profiling and functional analysis of intrauterine growth restriction (IUGR) piglets reveals a genetic correction and sexual-dimorphic gene expression during postnatal development. BMC Genomics 21:701. doi: 10.1186/s12864-020-07094-9, PMID: 33032518 PMC7545842

[ref7] Geng S. Zhang Y. Cao A. Liu Y. Di Y. Li J.. (2022). Effects of fat type and exogenous bile acids on growth performance, nutrient digestibility, lipid metabolism and breast muscle fatty acid composition in broiler chickens. Animals 12:1258. doi: 10.3390/ani12101258, PMID: 35625104 PMC9137457

[ref8] He Q. H. Ren P. P. Kong X. F. Xu W. X. Tang H. R. Yin Y. L.. (2011). Intrauterine growth restriction alters the metabonome of the serum and jejunum in piglets. Mol. BioSyst. 7, 2147–2155. doi: 10.1039/c1mb05024a, PMID: 21584308

[ref9] Hu J. Chen L. Zheng W. Shi M. Liu L. Xie C.. (2018). *Lactobacillus* *frumenti* facilitates intestinal epithelial barrier function maintenance in early-weaned piglets. Front. Microbiol. 9:897. doi: 10.3389/fmicb.2018.00897, PMID: 29867808 PMC5958209

[ref10] Huang S. Wu Z. Yuan X. Li N. Li T. Wang J. J.. (2020). Transcriptome differences suggest novel mechanisms for intrauterine growth restriction mediated dysfunction in small intestine of neonatal piglets. Front. Physiol. 11:561. doi: 10.3389/fphys.2020.00561, PMID: 32655399 PMC7324767

[ref11] Jiang L. Feng C. Tao S. Li N. Zuo B. Han D.. (2019). Maternal imprinting of the neonatal microbiota colonization in intrauterine growth restricted piglets: A review. J. Anim. Sci. Biotechnol. 10:88. doi: 10.1186/s40104-019-0397-7, PMID: 31737268 PMC6844051

[ref12] Kong X. F. Ji Y. J. Li H. W. Zhu Q. Blachier F. Geng M. M.. (2016). Colonic luminal microbiota and bacterial metabolite composition in pregnant Huanjiang mini-pigs: Effects of food composition at different times of pregnancy. Sci. Rep. 6:37224. doi: 10.1038/srep37224, PMID: 27917879 PMC5137017

[ref13] Lai W. Huang W. Dong B. Cao A. Zhang W. Li J.. (2018). Effects of dietary supplemental bile acids on performance, carcass characteristics, serum lipid metabolites and intestinal enzyme activities of broiler chickens. Poult. Sci. 97, 196–202. doi: 10.3382/ps/pex288, PMID: 29136214

[ref14] Lammasak K. Kijpakorn S. Angkanaporn K. (2019). Corrigendum to: Porcine bile powder supplementation of a high fat broiler diet in relation to growth performance and nutrient digestion. Anim. Prod. Sci. 59:1399. doi: 10.1071/AN18190_CO

[ref15] Liu Y. Azad M. A. K. Kong X. F. Zhu Q. Yu Z. G. (2022). Dietary bile acids supplementation modulates immune response, antioxidant capacity, glucose, and lipid metabolism in normal and intrauterine growth retardation piglets. Front. Nutr. 9:991812. doi: 10.3389/fnut.2022.991812, PMID: 36211492 PMC9534482

[ref16] Lombardo D. (2001). Bile salt-dependent lipase: Its pathophysiological implications. Biochim. Biophys. Acta 1533, 1–28. doi: 10.1016/s1388-1981(01)00130-5, PMID: 11514232

[ref17] Longo S. Borghesi A. Tzialla C. Stronati M. (2014). IUGR and infections. Early Hum. Dev. 90, S42–S44. doi: 10.1016/S0378-3782(14)70014-3 24709457

[ref18] Lucas L. N. Barrett K. Kerby R. L. Zhang Q. Cattaneo L. E. Stevenson D.. (2021). Dominant bacterial phyla from the human gut show widespread ability to transform and conjugate bile acids. mSystems 2021:e0080521. doi: 10.1128/mSystems.00805-21 PMC12338150 34463573

[ref19] Ma C. Gao Q. K. Zhang W. H. Azad M. A. K. Kong X. F. (2020). Alterations in the blood parameters and fecal microbiota and metabolites during pregnant and lactating stages in Bama mini-pigs as a model. Mediat. Inflamm. 2020:8829072. doi: 10.1155/2020/8829072, PMID: 33162832 PMC7607286

[ref20] Maisonnier S. Gomez J. Brée A. Berri C. Baéza E. Carré B. (2003). Effects of microflora status, dietary bile salts and guar gum on lipid digestibility, intestinal bile salts, and histomorphology in broiler chickens. Poult. Sci. 82, 805–814. doi: 10.1093/ps/82.5.805, PMID: 12762404

[ref21] Martinez-guryn K. Hubert N. Frazier K. Urlass S. Musch M. W. Ojeda P.. (2018). Small intestine microbiota regulate host digestive and absorptive adaptive responses to dietary lipids. Cell Host Microbe 23, 458–469.e5. doi: 10.1016/j.chom.2018.03.011, PMID: 29649441 PMC5912695

[ref22] Mcglone E. R. Bloom S. R. (2019). Bile acids and the metabolic syndrome. Ann. Clin. Biochem. 56, 326–337. doi: 10.1097/CM9.0000000000000696 30453753

[ref23] Metzler-zebeli B. U. Magowan E. Hollmann M. Ball M. E. E. Molnár A. Witter K.. (2018). Differences in intestinal size, structure, and function contributing to feed efficiency in broiler chickens reared at geographically distant locations. Poult. Sci. 97, 578–591. doi: 10.3382/ps/pex332, PMID: 29253222

[ref24] Meyer A. M. Hess B. W. Paisley S. I. Du M. Caton J. S. (2014). Small intestinal growth measures are correlated with feed efficiency in market weight cattle, despite minimal effects of maternal nutrition during early to midgestation. J. Anim. Sci. 92, 3855–3867. doi: 10.2527/jas.2014-7646, PMID: 25057033

[ref270] NRC (2012). Nutrient Requirements of Swine: Eleventh Revised Edition. Washington, DC: The National Academies Press. 420. doi: 10.17226/13298, PMID: 35120774

[ref25] Olszewski J. Zabielski R. Skrzypek T. Matyba P. Wierzbicka M. Adamski A.. (2021). Differences in intestinal barrier development between intrauterine growth restricted and normal birth weight piglets. Animals 11:990. doi: 10.3390/ani11040990, PMID: 33916133 PMC8065605

[ref26] Papizadeh M. Rohani M. Nahrevanian H. Javadi A. Pourshafie M. R. (2017). Probiotic characters of *Bifidobacterium* and *Lactobacillus* are a result of the ongoing gene acquisition and genome minimization evolutionary trends. Microb. Pathog. 111, 118–131. doi: 10.1016/j.micpath.2017.08.021, PMID: 28826768

[ref27] Su X. Yao B. (2022). Exploiting enzymes as a powerful tool to modulate the gut microbiota. Trends Microbiol. 30, 314–317. doi: 10.1016/j.tim.2022.01.003, PMID: 35120774

[ref28] Sun M. F. Shen Y. Q. (2018). Dysbiosis of gut microbiota and microbial metabolites in Parkinson's disease. Ageing Res. Rev. 45, 53–61. doi: 10.1016/j.arr.2018.04.004 29705121

[ref29] Tao S. Bai Y. Li T. Li N. Wang J. (2019). Original low birth weight deteriorates the hindgut epithelial barrier function in pigs at the growing stage. FASEB J. 33, 9897–9912. doi: 10.1096/fj.201900204RR, PMID: 31170357

[ref30] Tian L. Xu Y. Fu M. Peng T. Liu Y. Long S. (2011). The impact of plasma triglyceride and apolipoproteins concentrations on high-density lipoprotein subclasses distribution. Lipids Health Dis. 10:17. doi: 10.1186/1476-511X-10-17, PMID: 21251287 PMC3036640

[ref31] Verheyen A. J. Maes D. G. Mateusen B. Deprez P. Janssens G. P. De Lange L.. (2007). Serum biochemical reference values for gestating and lactating sows. Vet. J. 174, 92–98. doi: 10.1016/j.tvjl.2006.04.001, PMID: 16723263

[ref32] Wahlström A. Sayin S. I. Marschall H. U. Bäckhed F. (2016). Intestinal crosstalk between bile acids and microbiota and its impact on host metabolism. Cell Metab. 24, 41–50. doi: 10.1016/j.cmet.2016.05.005, PMID: 27320064

[ref33] Wang J. Chen L. Li D. Yin Y. Wang X. Li P.. (2008). Intrauterine growth restriction affects the proteomes of the small intestine, liver, and skeletal muscle in newborn pigs. J. Nutr. 138, 60–66. doi: 10.1093/jn/138.1.60, PMID: 18156405

[ref35] Wang K. Hu C. J. Tang W. Azad M. A. K. Zhu Q. He Q. H.. (2021). The enhancement of intestinal immunity in offspring piglets by maternal probiotic or synbiotic supplementation is associated with the alteration of gut microbiota. Front. Nutr. 8:686053. doi: 10.3389/fnut.2021.686053, PMID: 34307437 PMC8298840

[ref36] Wang M. Yang C. Wang Q. Y. Li J. Z. Li Y. L. Ding X. Q.. (2020). The growth performance, intestinal digestive and absorptive capabilities in piglets with different lengths of small intestines. Animal 14, 1196–1203. doi: 10.1017/S175173111900288X, PMID: 31829913

[ref37] Wu G. Bazer F. W. Wallace J. M. Spencer T. E. (2006). Board-invited review: Intrauterine growth retardation: Implications for the animal sciences. J. Anim. Sci. 84, 2316–2337. doi: 10.2527/jas.2006-156, PMID: 16908634

[ref38] Xiong L. You J. Zhang W. Zhu Q. Blachier F. Yin Y. L.. (2020). Intrauterine growth restriction alters growth performance, plasma hormones, and small intestinal microbial communities in growing-finishing pigs. J. Anim. Sci. Biotechnol. 11:86. doi: 10.1186/s40104-020-00490-x, PMID: 32832077 PMC7437023

[ref39] Zhang W. H. Ma C. Xie P. F. Zhu Q. Wang X. D. Yin Y. Y.. (2019). Gut microbiota of newborn piglets with intrauterine growth restriction have lower diversity and different taxonomic abundances. J. Appl. Microbiol. 127, 354–369. doi: 10.1111/jam.14304, PMID: 31077497 PMC6916403

[ref40] Zhao Y. Niu Y. He J. Gan Z. Ji S. Zhang L.. (2020). Effects of dietary dihydroartemisinin supplementation on growth performance, hepatic inflammation, and lipid metabolism in weaned piglets with intrauterine growth retardation. Anim. Sci. J. 91:e13363. doi: 10.1111/asj.13363, PMID: 32219939

[ref41] Zhu L. Baker R. D. Zhu R. Baker S. S. (2018). Bile acids and the gut microbiome as potential targets for NAFLD treatment. J. Pediatr. Gastroenterol. Nutr. 67, 3–5. doi: 10.1097/MPG.0000000000002010, PMID: 29697548

