# Supplementary material for: Dietary bile acid supplementation alters plasma biochemical and hormone indicators, intestinal digestive capacity, and microbiota of piglets with normal birth weight and intrauterine growth retardation
Source: Front Microbiol. 2022 Nov 10;13:1053128. doi: 10.3389/fmicb.2022.1053128 (PMC9684342; doi:10.3389/fmicb.2022.1053128)
Supplement: Supplementary file 1 [file Table_1.docx]

**Supplementary Table 1.** Ingredients and nutrient levels of a basal diet (%, as-fed basis)

| Items | Content |
| --- | --- |
| Ingredients | |
| Corn | 48.50 |
| Extruded soybean | 12.00 |
| Extruded corn | 10.00 |
| Soybean meal | 7.50 |
| Fermented soybean meal | 5.00 |
| Whey powder | 5.00 |
| Steam fish meal | 3.00 |
| Oil powder | 2.00 |
| Glucose | 2.00 |
| Premix ^1^ | 5.00 |
| Total | 100 |
| Nutrient levels ^2^ | |
| Digestible energy (MJ/kg) | 14.44 |
| Metabolizable energy (MJ/kg) | 13.81 |
| Crude protein | 17.50 |
| Crude fat | 4.70 |
| Ash | 4.10 |
| Crude fiber | 2.34 |
| Digestible sulfur-containing amino acids/lysine | 0.55 |
| Digestible threonine/lysine | 0.65 |
| Digestible tryptophan/lysine | 0.19 |

^1^ The premix provided the following per kilogram complete diet: vitamin A 12,000 IU; vitamin D_3_ 3,000 IU; vitamin E 50 mg; vitamin K_3_ 4 mg; vitamin B_1_ 4 mg; vitamin B_2_ 10 mg; vitamin B_6_ 7 mg; vitamin B_12_ 0.05 mg; acidifier 5.00 g; choline chloride 1.00 g; Cu (CuSO_4_ 5H_2_O) 0.50 g; Mn (MnSO_4_ H_2_O) 0.30 g; Zn (ZnSO_4_ H_2_O) 0.30 g; Fe (FeSO_4_ H_2_O) 0.60 g; I (KIO_3_) 10 mg; Se (Na_2_SeO_3_) 1% 10 mg.

^2^ Nutrient levels were calculated values.
